# Chemical composition and anti-cholesterol activity of tea (*Camellia sinensis*) flowers from albino cultivars

**DOI:** 10.3389/fnut.2023.1142971

**Published:** 2023-03-27

**Authors:** Ying Gao, Zhen Han, Yong-Quan Xu, Jun-Feng Yin

**Affiliations:** ^1^Key Laboratory of Tea Biology and Resources Utilization, Tea Research Institute Chinese Academy of Agricultural Sciences, National Engineering Research Center for Tea Processing, Hangzhou, China; ^2^Agro-Technical Extension Station of Ningbo City, Ningbo, China

**Keywords:** tea flowers, albino cultivars, chemical composition, anti-cholesterol, polyphenols, amino acids, saponins

## Abstract

Albino tea cultivars are mutant tea plants with altered metabolisms. Current studies focus on the leaves while little is known about the flowers. To evaluate tea flowers from different albino cultivars, the chemical composition and anti-cholesterol activity of tea flowers from three albino cultivars (i.e., Baiye No.1, Huangjinya, and Yujinxiang) were compared. According to the results, tea flowers from Yujinxiang had more amino acids but less polyphenols than tea flowers from the other two albino cultivars. A reduced content of procyanidins and a high chakasaponins/floratheasaponins ratio were characteristics of tea flowers from Yujinxiang. *In vitro* anti-cholesterol activity assays revealed that tea flowers from Yujinxiang exhibited stronger activity in decreasing the micellar cholesterol solubility, but not in cholesterol esterase inhibition and bile salt binding. It was noteworthy that there were no specific differences on the chemical composition and anti-cholesterol activity between tea flowers from albino cultivars and from Jiukeng (a non-albino cultivar). These results increase our knowledges on tea flowers from different albino cultivars and help food manufacturers in the cultivar selection of tea flowers for use.

## 1. Introduction

*Camellia sinensis* is a flowering plant in the Theaceae family. Its leaves have been used to produce tea, a globally popular beverage, for centuries. Therefore, *Camellia sinensis* is regarded as an important economic plant and widely cultivated. Tea flowers are reproductive organs of *Camellia sinensis*, usually white in color, blooming from September to December. The yield of tea flowers in matured tea gardens is about 2.86–4.29 tons/acre (1). Unlike tea leaves, tea flowers were under-evaluated in the past and mostly discarded as wastes. Recent researches have revealed that multiple bioactive compounds in tea leaves also exist in tea flowers (2). Compared with tea leaves, tea flowers contain more soluble sugars, but less polyphenols and caffeine (3). Evidences indicate that tea flowers display various bioactivities (2). Anti-cholesterol activity is a featuring activity of tea flowers. Cholesterol is a type of lipid which acts as an essential structural component of animal cell membranes and serves as precursor for bile acids, vitamin D, and steroid hormones (e.g., estrogen and testosterone) (4). However, too much cholesterol is harmful to the body, which is associated with the increased incidence of coronary artery diseases. Deng and Huang found that tea flowers exhibited anti-cholesterol activity *in vitro* (5). Ling et al. proved that oral administration of tea flowers significantly reduced the levels of serum total cholesterols and low density lipoproteins in high-fat diet-fed rats (6). Due to the excellent bioactivity and safety, tea flowers were approved as novel food components by the National Health Commission of the People’s Republic of China in 2013, which permitted the legal application of tea flowers in commercial foods. By now, tea flowers have been used in beverages and snacks to improve the health-beneficial function (7, 8).

The health-beneficial function of tea flowers is determined by the chemical components. It has been reported that the chemical components in tea flowers are affected by the floral organ, flowering stage, growth area, and cultivar (3, 9–11). The cultivar not only has an impact on the levels of general components (e.g., catechins), but also on the presence of specific components (e.g., anthocyanins) (12).

Albino tea cultivars are mutant tea plants whose leaves are notably lighter in color compared with non-albino tea leaves due to the lack of chlorophylls (13). The balance of carbon and nitrogen metabolism is hampered in albino leaves, resulting in the accumulation of free amino acids and the shortage of polyphenols (14), which makes albino tea taste less bitter and astringent than non-albino tea. The synthesis of volatiles in albino tea leaves is also altered, leading to a more ethereal smell (15). Currently, more attentions are paid on studying the leaves than flowers of albino tea plants. Little is known about tea flowers from albino cultivars. In this study, the chemical composition and anti-cholesterol activity of tea flowers from three albino cultivars (i.e., Baiye No. 1, Huangjinya, and Yujinxiang) and one traditional non-albino cultivar (i.e., Jiukeng) were investigated. The results will enhance our knowledge on tea flowers from different cultivars and guide food producers choose the proper tea flowers for applications.

## 2. Materials and methods

### 2.1. Tea flower samples and reagents

Dried tea flower samples were provided by Yuyao Sichuangyan Tea Co. Ltd (Zhejiang, China). The tea flowers were collected at the half-open period from the same tea garden in Yuyao City, Zhejiang Province, China, in November. Tea flowers were withered for 4 h, dried by hot air at 40°C for 1 h (Hot air dryer, Product No. 6CHZ-9B, Fujian Jiayou tea machinery Co., Ltd., Fujian, China), cooled for 30 min, dried by hot air at 55°C for 1 h and at 70°C for 1 h to obtain dried tea flowers. The weight of fresh tea flowers in each batch was 50 kg. Three batches of samples were prepared for each cultivar.

Sodium cholate, sodium taurocholate, sodium glycocholate, and sodium chloride were purchased from Shanghai Macklin Biochemical Technology Co., Ltd (Shanghai, China). Cholesterol, cholesterol esterase, 4-nitrophenyl butyrate, cholestyramine, and epigallocatechin gallate (EGCG) were purchased from Shanghai Yuanye Bio-Technology Co., Ltd (Shanghai, China). Oleic acid was purchased from Sigma-Aldrich (Shanghai) Trading Co., Ltd (Shanghai, China).

### 2.2. Chemical composition analysis

Each tea flower sample was ground and filtered through a 60 Tyler mesh sieve. The tea flower powder was extracted with distilled water (3:500 w/v) at 100°C for 40 min and centrifuged at 8,000 g for 10 min to remove powder residues. The supernatant was used for analysis.

The total phenolic content was measured according to the national standard GB/T 8313–2008. The contents of eight monomeric catechins were measured using a published HPLC method (16). The content of free amino acids was measured according to the national standard GB/T 8314–2013. The composition of free amino acids was measured by an amino acid analyzer (Sykam S433-D, Eresing, Germany) using a previous method (17). Detailed description of each method was presented in the supplementary material.

The content of soluble sugars was measured using anthrone-sulfuric acid method. In brief, 1 mL sample solution was mixed with 4 mL anthrone-sulfuric acid, incubated at 100°C for 10 min, cooled to room temperature, and then measured the absorbance at 620 nm. To measure the soluble polysaccharide content, the polysaccharide was precipitated by mixing the sample solution with ethanol at a ratio of 1:5, cooled at 4°C overnight, and centrifuged at 8,500 g for 10 min. The precipitate was re-dissolved with distilled water to prepare the solution for the anthrone-sulfuric acid assay. The contents of sucrose, fructose, and glucose were determined using corresponding commercial kits (Beijing Solarbio Science & Technology Co., Ltd., Products No. BC2465, BC2455, and BC2500, Beijing, China).

The content of saponins was measured using the vanillin-sulfuric acid method. A 100 μL sample solution was mixed with 0.5 mL 8% vanillin-alcohol solution and 3 mL 70% sulfuric acid, incubated at 60°C for 10 min, cooled to room temperature, and read the absorbance at 540 nm.

Ultrahigh performance liquid chromatography-Q Exactive-Orbitrap-mass spectrometry (UPLC-QE-Orbitrap-MS) was used to determine the composition of procyanidins, organic acids, flavonols, methylxanthines, and saponins, based on published methods with modifications (11, 18). The separation was performed on an ACQUITY UPLC BEH C18 column (1.7 μm, 2.1 mm × 100 mm, Waters, Milford, MA, United States) using a Dionex Ultimate 3,000 RS system. A 0.1% formic acid in water and acetonitrile was used as mobile phases A and B. To determine procyanidins, methylxanthines, and organic acids, the gradient changes of mobile phases were 0–1 min, 5% B; 1–2 min, 5–10% B; 2–6 min, 10–35% B; 6–8.5 min, 35–100% B; 8.5–9.5 min, 100% B; 9.5–10 min, 100–5% B; and 10–12 min, 5% B. To determine saponins, the gradient changes of mobile phases were 0–5 min, 25–35% B; 5–6 min, 35–37% B; 6–15 min, 37–45% B; 15–18 min, 45–100% B; 18–19 min, 100% B; 19–20 min, 100–25% B; and 20–22 min, 25% B. To determine flavonols, the gradient changes of mobile phases were 0–6 min, 10–30% B; 6–9.5 min, 30–100% B; 9.5–10 min, 100–10% B; 10–12 min, 10% B. The total flow rate was 0.3 mL/min. The column temperature was 40°C. The MS analysis was conducted using the QE-Orbitrap mass spectrometer (Thermo Scientific, United States) with electrospray ionization (ESI), operating in the positive and negative ionization full scan modes. Auxiliary gas and sheath gas flow rates were 10 and 45 (arbitrary units), respectively. The auxiliary gas heater temperature was 300°C. The capillary temperature was 320°C. The spray voltage was 3.1 kV and the S-lens RF level was 50 V. The normalized collision energy (NCE) was 30 eV. The resolution of the full scan and ddMS2 were 70,000 and 35,000, respectively. The full MS scan ranges were set from m/z 100 to 1,500. Data were acquired and processed using ThermoXcalibur 3.0 software (Thermo Scientific, United States). Tentative identification was based on comparing retention time, m/z values, and MS/MS fragments with standards or data from databases (e.g., MzCloud) when standards were unavailable (Detailed information is provided in Supplementary Table S1 and Supplementary Figure S1). Relative quantitation was calculated by comparing the relative intensities of the parent ions among samples and presented in a heat map after converting to Z-scores of the rows.

### 2.3. Micellar cholesterol solubility assay

The assay was conducted based on a previously published method (19). The micellar cholesterol solution consisted of 2 mmol/L cholesterol, 10 mmol/L sodium taurocholate, 5 mmol/L oleic acid, 132 mmol/L sodium chloride, and 15 mmol/L phosphate buffered saline (0.1 mol/L, pH = 7.4). One hundred microliter tea flower solution (or distilled water as the control) was mixed with 900 μL micellar cholesterol solution. One hundred microliter tea flower solution was mixed with 900 μL distilled water as the sample control. The mixture was incubated at 37°C for 1 h and centrifuged at 15,000 g for 20 min. The cholesterol concentration of the supernatant was measured using a total cholesterol assay kit (Nanjing Jiancheng Bioengineering Institute, Product No. A111-1-1, Nanjing, China). The remaining cholesterol (%) was calculated as follows.


$$\mathrm{Remaining cholesterol}\;\left(\%\right)=\frac{A_{\mathrm{sample}}-A_{\mathrm{sample control}}}{A_{\mathrm{control}}}\times 100\%$$

### 2.4. Cholesterol esterase inhibition assay

The assay was conducted based on a previously published method (20). One milliliter phosphate buffered saline (0.1 mol/L, pH = 7.0) containing 5.16 mmol/L sodium taurocholate and 0.1 mol/L sodium chloride was mixed with 50 μL cholesterol esterase (5 mU/mL), 20 μL 4 mmol/L 4-nitrophenyl butyrate, and 50 μL tea flower infusion (or 50 μL distilled water as the control). One milliliter phosphate buffered saline was mixed with 50 μL distilled water, 20 μL 4 mmol/L 4-nitrophenyl butyrate, and 50 μL tea flower infusion as the sample control. The mixture was incubated at 25°C for 30 min. The absorbance at 405 nm was measured and recorded as A_sample_ (or A_control_). The inhibition rate was calculated using the following formula.


$$\mathrm{Inhibition}\;\left(\%\right)=\frac{A_{\mathrm{control}}-\left(A_{\mathrm{sample}}-A_{\mathrm{sample control}}\right)}{A_{\mathrm{control}}}\times 100\%$$

### 2.5. Bile salt binding assay

The assay was conducted based on a previously published method (5) with some modifications. One hundred microliter tea flower infusion (or distilled water as the control) was mixed with 400 μL 0.5 mmol/L bile salt (sodium cholate, sodium taurocholate, or sodium glycocholate). One hundred microliter tea flower infusion was mixed with 400 μL distilled water as the sample control. and incubated at 37°C for 1 h. The mixture was centrifuged at 4,000 g for 20 min to obtain the supernatant. Two hundred microliter supernatant was mixed with 600 μL 60% sulfuric acid, incubated at 70°C for 20 min. After cooling to room temperature, the absorbance at 387 nm was determined. The bile salt binding rate was calculated as follows.


$$\begin{array}{l} \mathrm{Bile salt binding rate}\;\left(\%\right)\\ =\frac{A_{\mathrm{control}}-\left(A_{\mathrm{sample}}-A_{\mathrm{sample control}}\right)}{A_{\mathrm{control}}}\times 100\% \end{array}$$

### 2.6. Statistical analysis

The data are presented as the mean ± standard error of the mean (SEM). All experiments were carried out in triplicate and repeated in three independent sets of experiments. The results were analyzed with SPSS Version 18.0 for Windows using the one-way analysis of variance with *post hoc* test (2-sided Dunnett’s test). The difference was deemed significant if the value of *p* was less than 0.05. Partial least squares regression was performed using the SIMCA 13.0 software.

## 3. Results and discussion

### 3.1. Chemical composition

General chemical analysis (Table 1) revealed that soluble sugars were the most abundant component in tea flowers, accounting for about 40%. Saponins were the second most abundant component, occupying over 10%. Polyphenols listed the third.

**Table 1:** The chemical compositions of four tea flowers.

Content (mg/g)	Baiye No.1	Huangjinya	Yujinxiang	Jiukeng
Free amino acids	27.74 ± 0.44^b^	24.10 ± 0.95^c^	36.35 ± 0.32^a^	36.14 ± 0.44^a^
Polyphenols	78.03 ± 1.17^b^	83.21 ± 2.55^a^	70.90 ± 0.56^c^	77.03 ± 0.22^b^
Soluble sugars	414.95 ± 31.19^a^	400.74 ± 16.11^a^	449.30 ± 16.87^a^	436.37 ± 22.31^a^
Soluble polysaccharides	14.23 ± 0.12^b^	13.17 ± 0.51^b^	16.19 ± 1.21^a^	12.40 ± 0.68^b^
Saponins	122.11 ± 2.37^ab^	135.02 ± 4.83^a^	115.76 ± 3.35^b^	120.84 ± 2.44^ab^

The same letter within each row indicates no significant difference (*p* > 0.05).

The content of soluble sugars was insignificantly different among the four samples, ranging from 400.74 to 449.30 mg/g. Xu et al. revealed that the content of soluble sugars in Longjing No.43 (a non-albino cultivar) tea flowers from Zhejiang Province was 422.73 mg/g (21). It provided more evidence that the content of soluble sugars was similar among cultivars. Sucrose, fructose, and glucose were major soluble sugars in tea flowers (Table 2), which was consistent with a previous study (21). The three small molecular sugars together made up about 70% of total soluble sugars. Baiye No.1, a temperature-sensitive albino cultivar., contained the least sucrose but the most glucose among the four samples. Though both belonging to light-sensitive albino cultivars, Yujinxiang had a higher glucose content than Huangjinya. The content of fructose was insignificantly different. Soluble polysaccharides, which were macromolecules contributing to the thick taste of tea infusions and displayed multiple bioactivities such as antioxidant and α-glucosidase inhibitory activity (18, 22), were observed in tea flowers, ranging from 12.4 to 16.2 mg/g (Table 1). Yujinxiang had a higher content of soluble polysaccharides compared with the other three. Huang et al. previously demonstrated that the content of soluble polysaccharides in tea flowers from non-albino cultivars ranged from 1.54 to 2.34% (23), most of which were higher than our results. It implied that the content of soluble polysaccharides was affected by the cultivar.

**Table 2:** The contents of soluble sugars, monomeric catechins, procyanidins, free amino acids, organic acids, and methylxanthines in four tea flowers.

Contents	Baiye No.1	Huangjinya	Yujinxiang	Jiukeng
Soluble sugars (mg/g)				
Sucrose	88.38 ± 4.77^c^	107.32 ± 7.17^b^	114.90 ± 9.53^b^	137.63 ± 1.09^a^
Fructose	104.67 ± 5.09^a^	96.15 ± 8.17^a^	125.24 ± 24.14^a^	90.15 ± 10.67^a^
Glucose	89.62 ± 2.04^a^	75.30 ± 3.66^b^	90.43 ± 1.69^a^	79.08 ± 0.94^b^
Monomeric catechins (mg/g)				
Gallocatehin (GC)	1.91 ± 0.11^d^	4.75 ± 0.12^a^	3.07 ± 0.03^c^	4.06 ± 0.04^b^
Epigallocatechin (EGC)	1.82 ± 0.14^a^	1.39 ± 0.04^a^	1.46 ± 0.06^a^	1.10 ± 0.02^b^
Catechin (C)	0.99 ± 0.14^a^	1.15 ± 0.04^a^	0.15 ± 0.02^c^	0.80 ± 0.03^b^
Epigallocatechin gallate (EGCG)	8.89 ± 0.35^c^	7.11 ± 0.17^d^	12.96 ± 0.14^a^	9.81 ± 0.09^b^
Epicatechin (EC)	4.89 ± 0.18^a^	4.17 ± 0.19^b^	2.57 ± 0.31^d^	3.61 ± 0.05^c^
Gallocatechin gallate (GCG)	1.93 ± 0.09^c^	1.30 ± 0.03^d^	2.30 ± 0.03^a^	2.09 ± 0.04^b^
Epicatechin gallate (ECG)	4.05 ± 0.10^b^	5.19 ± 0.12^a^	5.01 ± 0.04^a^	5.01 ± 0.05^a^
Catechin gallate (CG)	0.37 ± 0.01^c^	0.48 ± 0.02^b^	0.51 ± 0.02^b^	0.56 ± 0.01^a^
Total monomeric catechins	24.85 ± 1.03^c^	25.53 ± 0.70^bc^	28.02 ± 0.44^a^	27.03 ± 0.20^ab^
Procyanidins (mg/g)				
Procyanidin B1	1.36 ± 0.01^c^	2.19 ± 0.01^a^	0.28 ± 0.01^d^	1.49 ± 0.03^b^
Procyanidin B2	5.10 ± 0.08^b^	7.51 ± 0.06^a^	1.50 ± 0.08^d^	4.56 ± 0.19^c^
Procyanidin C1	0.54 ± 0.03^b^	0.82 ± 0.05^a^	0.33 ± 0.02^c^	0.48 ± 0.04^b^
Free amino acids (μg/g)				
Aspartate	112.7 ± 4.2^a^	55.9 ± 1.6^c^	46.8 ± 3.3^d^	63.7 ± 1.8^b^
Threonine	163.4 ± 4.0^b^	90.9 ± 6.9^d^	189.1 ± 7.3^a^	117.4 ± 9.0^c^
Serine	799.6 ± 4.9^a^	373.7 ± 1.1^d^	605.6 ± 12.0^b^	456.7 ± 19.2^c^
Asparagine	181.4 ± 8.1^a^	130.3 ± 4.3^b^	135.2 ± 3.0^b^	171.9 ± 8.1^a^
Glutamic acid	707.2 ± 14.7^a^	462.9 ± 8.6^c^	428.9 ± 14.7^d^	494.1 ± 7.2^b^
Theanine	2914.4 ± 50.1^c^	1339.8 ± 11.4^d^	4124.5 ± 125.7^b^	5357.4 ± 158.3^a^
Proline	1124.8 ± 25.7^b^	1173.2 ± 38.2^b^	1580.9 ± 21.2^a^	1503.7 ± 64.3^a^
Glycine	42.3 ± 2.4^a^	<LOQ^c^	29.7 ± 2.8^b^	23.2 ± 4.6^b^
Alanine	1000.7 ± 15.9^a^	595.6 ± 7.3^c^	677.0 ± 29.3^b^	580.2 ± 12.7^c^
Valine	187.2 ± 36.9^a^	126.6 ± 7.1^a^	105.5 ± 8.2^a^	155.1 ± 21.2^a^
Methionine	179.5 ± 6.9^b^	263.0 ± 3.3^ab^	271.8 ± 13.1^ab^	362.2 ± 21.8^a^
Isoleucine	69.7 ± 7.3^a^	40.0 ± 2.2^b^	49.5 ± 4.7^ab^	42.5 ± 0.5^ab^
Leucine	41.9 ± 4.4^a^	30.7 ± 2.4^a^	19.1 ± 1.3^a^	31.9 ± 3.2^a^
γ-Aminobutyric acid	340.9 ± 10.1^a^	288.6 ± 7.7^b^	218.3 ± 12.6^c^	338.4 ± 0.9^a^
Histidine	64.3 ± 1.4^c^	66.3 ± 1.3^bc^	70.7 ± 1.4^b^	99.8 ± 4.3^a^
Ornithine	27.9 ± 5.3^a^	35.0 ± 3.2^a^	31.4 ± 2.4^a^	31.4 ± 4.6^a^
Lysine	48.3 ± 1.2^a^	54.4 ± 6.6^a^	44.0 ± 2.2^b^	51.1 ± 4.2^a^
Arginine	150.2 ± 3.3^b^	358.1 ± 8.6^a^	348.8 ± 8.8^a^	440.1 ± 33.4^a^
Organic acids (mg/g)				
Quinic acid	2.18 ± 0.02^a^	1.69 ± 0.27^b^	1.05 ± 0.03^c^	1.59 ± 0.02^b^
Gallic acid	4.95 ± 0.36^a^	2.83 ± 0.15^b^	4.23 ± 0.26^a^	3.93 ± 0.21^a^
Citric acid	15.16 ± 0.72^a^	9.99 ± 0.32^c^	12.25 ± 0.91^b^	14.55 ± 0.40^a^
Malic acid	8.40 ± 0.29^a^	7.46 ± 0.47^b^	7.37 ± 0.15^b^	7.52 ± 0.16^b^
Succnic acid	1.13 ± 0.01^c^	1.70 ± 0.04^a^	1.70 ± 0.05^a^	1.51 ± 0.09^b^
Methylxanthines (μg/g)				
Caffeine	4966.9 ± 124.3^a^	4453.5 ± 122.4^b^	3291.7 ± 69.8^d^	4022.5 ± 20.1^c^
Theobromine	157.7 ± 6.5^a^	55.7 ± 4.4^d^	84.8 ± 3.3^c^	97.4 ± 4.2^b^
Theophylline	9.3 ± 0.8^a^	10.4 ± 1.0^a^	4.5 ± 0.5^b^	5.2 ± 0.2^b^

The same letter within each row indicates no significant difference (*p* > 0.05). LOQ is short for the limit of quantification.

**Figure 1:**
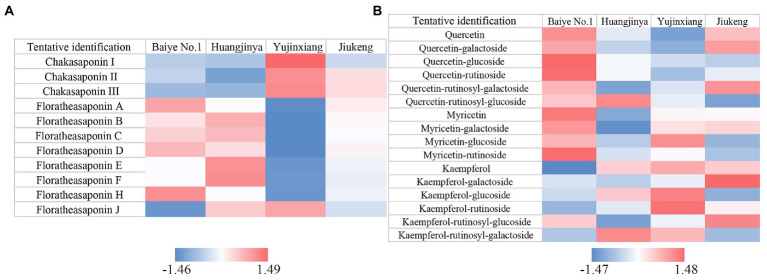
Heatmaps of Z-score normalized relative abundances of **(A)** saponins and **(B)** flavonols identified by UPLC-QE-Orbitrap-MS.

The content of saponins was varied among the four samples, ranging from 115.76 to 135.02 mg/g (Table 1). Huangjinya had the highest, while Yujinxiang had the least. The content of saponins in tea flowers was comparable to that in *Panax ginseng C. A. Meyer* flowers (24) and in *Panax Notoginseng* flowers (25), two plants which were abundant in saponins, indicating that tea flowers might be a good source of plant-derived saponins. UPLC-QE-Orbitrap-MS analysis revealed that the composition of saponins was distinct, especially between Yujinxiang and the other three samples (Figure 1A). The contents of chakasaponins I, II, and III were highest in Yujinxiang while lowest in Huangjinya. In contrast, the content of floratheasaponins was lowest in Yujinxiang. Floratheasaponins B, E, and F were accumulated in Huangjinya. Floratheasaponins A, D, and H were accumulated in Baiye No.1. A previous study indicated that distinct regional difference was observed with respect to the content of chakasaponins and floratheasaponins. The content of chakasaponins was higher in the extracts of tea flowers from Fujian and Sichuan provinces, China than those from Japan, Taiwan, and India (26). Shen et al. found that floratheasaponins were more abundant than chakasaponins in tea flowers from Jiaming No.1, Longjing No.43, and Baiye No.1 (11). On the contrary, chakasaponins were more abundant than floratheasaponins in tea flowers from Yingshuang and Fudingdabai. Our results confirmed that the abundances of floratheasaponins and chakasaponins in tea flowers were affected by the cultivar. Previous studies indicated that the activity of each saponin monomer was not the same. For example, floratheasaponins A-F, particularly floratheasaponins B & E, displayed strong anti-allergic activity by inhibiting the release of β-hexosaminidase from RBL-2H3 cells (27). Chakasaponins I-III inhibited the increase of plasma triglycerides and glucose partially by preventing gastric emptying (28). It was possible that the differential composition and content of saponins might lead to differential bioactivity among cultivars.

The content of polyphenols ranged from 70.90 to 83.21 mg/g among the four samples, with the lowest content found in Yujinxiang (Table 1). Previous studies showed that the content of polyphenols in tea flowers from non-albino cultivars collected in Guangxi and Yunnan Province ranged from 9.17 to 13.56% (29, 30), which was much higher than that we observed in tea flowers from albino cultivars. However, in our study, the content of polyphenols in tea flowers from Jiukeng was not higher than that from Baiye No.1 and Huangjinya. The inconsistency might be caused by the fact that not only the cultivar but also the region affected the content of polyphenols (29, 30).

Eight monomeric catechins were observed in tea flowers. EGCG, ECG, and EC were the three most abundant ones. The monomeric catechin composition of the four tea flower samples was distinguishable (Table 2). Yujinxiang had the highest EGCG and GCG contents among them. Compared with Yujinxiang, the GC and C levels were higher in Huangjinya. Baiye No.1 had a higher EC level than the others. Besides monomeric catechins, procyanidin B1 and B2, belonging to dimeric catechins, were observed in tea flowers (Table 2). Procyanidin B2 was the predominant one, ranging from 1.50 to 7.51 mg/g, while the content of procyanidin B1 ranged from 0.28 to 2.19 mg/g. Tea flowers from Huangjinya had more procyanidin B2 and B1 than the others. The procyanidin B2 content in Huangjinya was five times as much as that in Yujinxiang. As for procyanidin B1, the level in Huangjinya was about 8.1-fold of that in Yujinxiang. The results demonstrated that the contents of dimeric catechins were quite different among the four samples. A similar trend was observed in the content of procyanidin C1 (Table 2). Monomeric catechins and procyanidins are star molecules in plants. As secondary metabolites, they protect the plants from microbial infection and high intensity light-induced stress (31, 32). As well-known health-beneficial components, they not only possess excellent antioxidant activity, but also are involved in the regulations of energy metabolisms, immunity, and gut microbiota (33). The differences on monomeric catechins and procyanidins among the four samples might lead to the differences on the bioactivity. Besides, monomeric catechins and procyanidins are typical astringent compounds, which play important roles in the taste of grape wine and tea (34, 35), implying that they may affect the taste of tea flowers as well.

Based on previous findings and our results (29, 30), the ratio of catechins/polyphenols in tea flowers was much lower than that in tea leaves, suggesting the presence of other categories of polyphenols. Flavonols are a class of flavonoids that have the 3-hydroxyflavone backbone. Many of them possess antioxidant capacity, anti-inflammatory activity, and antimicrobial activity (36). A former study revealed that flavonols mainly existed in the forms of monoglycosides, diglycosides, and triglycosides in tea flowers, with quercetin, myricetin, and kaempferol as the main aglycones (37). In this study, five quercetin glycosides, three myricetin glycosides, and five kaempferol glycosides were observed in tea flowers (Figure 1B). Compared with the other three cultivars, Baiye No.1 contained higher contents of quercetin, quercetin glucoside, quercetin rutinoside, myricetin, myricetin galactoside, and myricetin rutinoside, but less kaempferol and derivatives. Yujinxiang contained less quercetin and derivatives, while Huangjinya contained less myricetin and myricetin galactoside. The above results revealed that the composition of flavonols in tea flowers was cultivar-specific, which was in accordance with the previous findings in tea flowers from non-albino cultivars (12).

Amino acids participate in extensive biochemical process in the body (38). Accumulated amino acids is a typical characteristic of albino tea leaves (14). Enhanced theanine synthesis and reduced amino acid transport are found in albino tea leaves (39). In this study, 18 amino acids were observed in tea flowers, including 15 proteinogenic amino acids and 3 non-protein amino acids (i.e., theanine, γ-aminobutyric acid, and ornithine) (Table 2). Six of them belonged to essential amino acids, including threonine, valine, methionine, isoleucine, leucine, and lysine. These essential amino acids accounted for about 7.4–11.0% of total amino acids in the four tea flower samples. The percentage of essential amino acids in tea flowers from Huangjinya was higher than that in other three samples. Besides the nutritional value, some amino acids play dual roles in the taste and health benefits. For example, theanine is the key umami compound in tea and shows potentials in regulating the nervous system and multiple metabolisms (40). Theanine was the most abundant amino acids in tea flowers. It was unexpected that tea flowers from Jiukeng had a higher theanine content than the other three, implying that the theanine metabolism was not abnormal in tea flowers from albino cultivars. Among the three albino samples, the theanine content in Yujinxiang was three times as much as that in Huangjinya. Proline was the second most abundant amino acid. Tea flowers from Yujinxiang had about 40% more proline than that from Baiye No.1 and Huangjinya.

Citric acid, malic acid, gallic acid, quinic acid, and succinic acid were organic acids observed in tea flowers (Table 2), all of which were taste compounds. Citric acid, malic acid, and succinic acid have sour taste and are widely used as food ingredients to modify the flavor. Gallic acid and quinic acid have astringent taste. Compared with tea leaves (41), the levels of organic acids in tea flowers were much lower. Among the four samples, tea flowers from Baiye No.1 had a higher level of organic acids than others. The organic acid composition of each tea flower sample was varied, indicating the cultivar-specificity.

Methylxanthines are secondary metabolites derived from purine nucleotides and widely distributed in plants such as *Camellia sinensis* (42). Caffeine plays a role in plant defenses against abiotic and biotic stresses (43). In animals and humans, caffeine acts as a central nervous system stimulant, theobromine is used as a mild cardiac stimulant and vasodilator, and theophylline is used as a bronchodilator. The three methylxanthines were observed in these flower samples. Among them, caffeine was the major one, ranging from 3.29 to 4.97 mg/g. Yujinxiang possessed the lowest caffeine content, while Baiye No.1 had the highest. Compared with tea leaves, the caffeine content in tea flowers was much lower, indicating that tea flowers might be suitable for caffeine-sensitive consumers. Theobromine was the second abundant methylxanthine, ranging from 55.7 to 157.7 μg/g. The theobromine content in Huangjinya was almost three times as much as that in Baiye No.1. A trace of theophylline was also detected. A previous research indicated that the caffeine and theobromine contents in tea flowers from non-albino cultivars collected in Chongqing city were 1.89 to 4.94 mg/g and 103.0 to 169.9 μg/g, respectively (44). It implied that the cultivar rather than the region might have a more profound impact on the caffeine and theobromine contents.

These data suggested that the differences of tea flowers between albino cultivars and Jiukeng (a normal cultivar) on the chemical composition were not as much as expected. Reduction of polyphenols and accumulation of amino acids, which were representative characteristics of albino tea leaves, were not obvious in tea flowers from albino cultivars. Nevertheless, the content and composition of saponins, catechins, procyanidins, flavonols, amino acids, organic acids, and methylxanthines in tea flowers were varied among albino cultivars, which might lead to differential health-beneficial functions.

### 3.2. Anti-cholesterol activity

Previous researches demonstrated the effectiveness of tea flowers on anti-cholesterol activity *in vitro* and *in vivo* (5, 6). Absorption of dietary cholesterol is an important part of cholesterol homeostasis (45). It has been proved that the rate of intestinal cholesterol absorption is related to pancreatic cholesterol esterase activity (46). Inhibition of pancreatic cholesterol esterase effectively reduces cholesterol absorption *in vivo* (47). Bile salts not only protect cholesterol esterase against proteolytic inactivation (48), but also promote the emulsification of cholesterol in the intestinal lumen and increase the absorption of cholesterol by forming micelles (49). Bile salts are produced in hepatocytes by converting cholesterol, stored in the gallbladder, and released to the duodenum *via* bile ducts. The majority of bile salts are reabsorbed from the intestine and pass back to the liver. When the process is interrupted, the liver consumes more cholesterol to compensate the loss. Cholestyramine, a medication used to treat hyperlipidemia, lowers the serum cholesterol level by forming nonabsorbable complex with bile salts in the intestinal lumen and disturbing the enterohepatic circulation of bile salts, which promotes hepatocytes convert more cholesterol to produce bile salts. In this study, three *in vitro* assays were conducted to compare the anti-cholesterol activity of the four samples.

**Figure 2:**
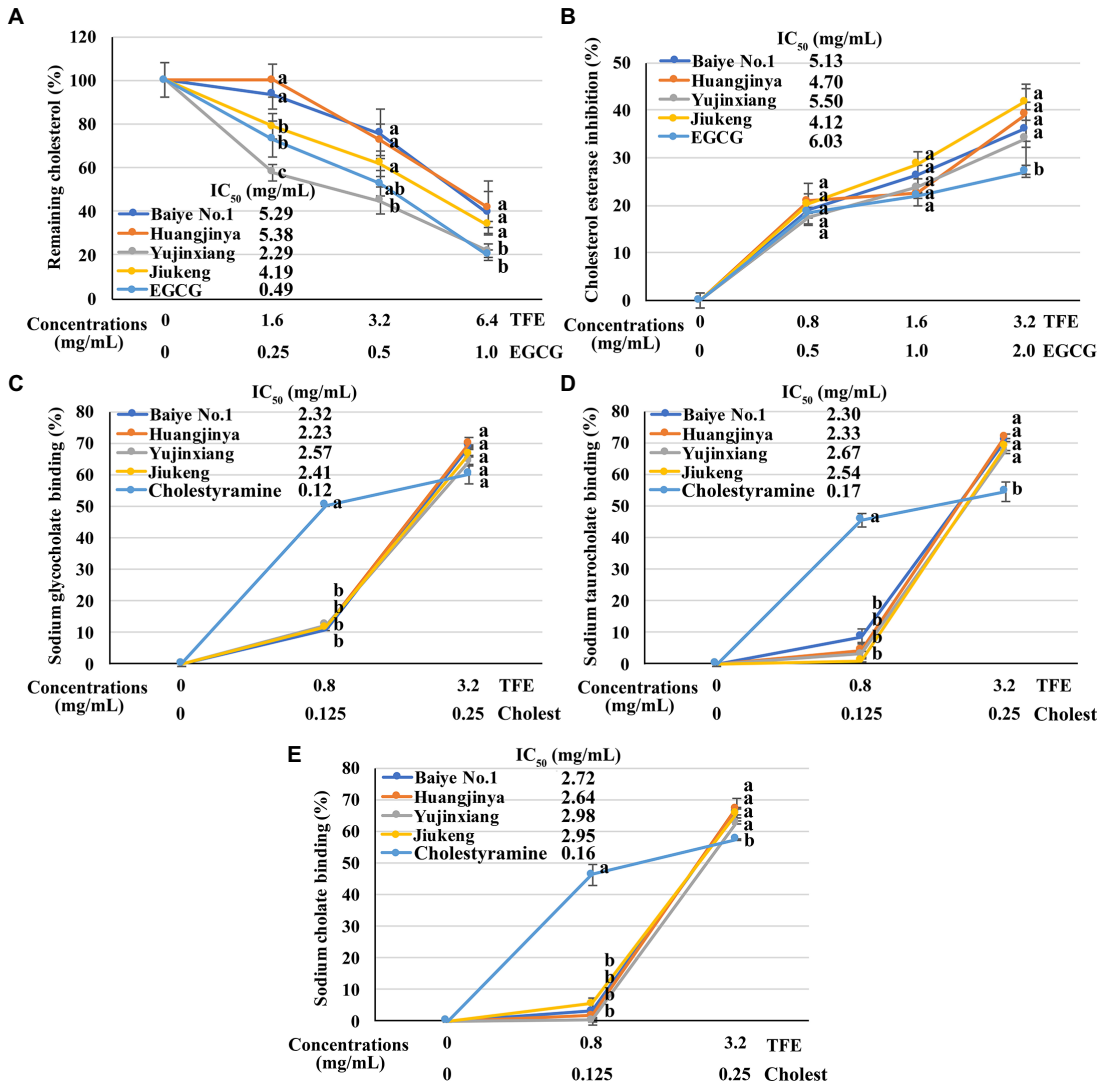
The *in vitro* anti-cholesterol activities of four tea flowers. **(A)** The activity of decreasing the micellar cholesterol solubility. **(B)** The cholesterol esterase inhibitory activity. **(C–E)** The sodium glycocholate **(C)**, sodium taurocholate **(D)**, sodium cholate **(E)**, and binding activity. Epigallocatechin gallate (EGCG) was used as the positive control for the micellar cholesterol solubility assay and cholesterol esterase inhibitory assay. Cholestyramine (Cholest) was used as the positive control for the bile salt binding assays. TFE is short for the tea flower extract. IC_50_ is short for the half maximal inhibitory concentration. The same letter within each row indicates no significant difference (*p* > 0.05).

All the four tea flower samples dose-dependently decreased the cholesterol solubility in a micellar solution. Among them, tea flowers from Yujinxiang were more capable than the other three (Figure 2A). The remaining cholesterol concentration in the solution exposed to Yujinxiang tea flower infusion was the lowest. The ability of the other three tea flower samples on decreasing the cholesterol solubility were comparable. It was previously revealed that tea polyphenols and polysaccharides decreased micellar cholesterol solubility (50, 51), while saponins enhanced it due to the surface activity (52). Although tea flowers from Yujinxiang contained less polyphenols, they contained less saponins and more polysaccharides, which might be advantageous to the decrease of micellar cholesterol solubility.

All the four tea flowers dose-dependently inhibited the activity of cholesterol esterase. However, the inhibitory effects of the four samples were insignificantly different (Figure 2B). It was reported that gallic acid, quercetin, and monomeric catechins were pancreatic cholesterol esterase inhibitors (53, 54). Compared with EGCG, the half maximal inhibitory concentration of quercetin was smaller (54). According to chemical analysis, tea flowers from Yujinxiang contained more monomeric catechins, while tea flowers from Baiye No.1 contained more quercetin. The presence of multiple compounds with differential cholesterol esterase inhibitory activity and their differed contents among samples might lead to the indifferent cholesterol esterase inhibitory activity.

The binding activity of tea flowers with three primary bile salts, i.e., sodium taurocholate, sodium glycocholate, and sodium cholate, was investigated. Each tea flower sample exhibited bile salt binding activity. The binding efficiency of tea flowers to each bile salt was similar and the binding activity of each tea flower sample to the same bile salt was not significantly different (Figures 2C–E). Deng et al. found that the bile salt binding activity of tea flowers was correlated to the total polyphenol content (5). Further study revealed that not only polyphenols, but also polysaccharides and caffeine had bile salt binding activity. The bile salt binding activity of polyphenols was stronger than that of polysaccharides and caffeine (55). Soluble proteins in tea flowers had affinity to three primary bile salts and showed the potential of anti-cholesterol activity *in vitro* (56). A recent research suggested that some saponins also had bile salt binding activity (57). In this study, insignificant differences on the bile salt binding activity of the four tea flowers were observed, probably due to the differences on the content and composition of active bile salt binding components in each tea flower sample.

The above results demonstrated that tea flowers from Yujinxiang exhibited better activity in decreasing micellar cholesterol solubility, while the four samples showed no significant differences on the cholesterol esterase inhibition and bile salt binding.

To figure out the relationship between chemical composition and anti-cholesterol activity, partial least squares regression analysis was performed. The contents of 45 compounds were set as X variables, while the cholesterol reduction (%), cholesterol esterase inhibition (%), and the binding rates of three bile salts were set as Y variables. According to the results (Supplementary Figure S2), 21 compounds with variable importance in projection (VIP) >1 were screened out. Among them, 7 compounds, including EGCG and chakasaponins I-III, were positively correlated to the anti-cholesterol activity. While 14 compounds, including several floratheasaponins, were negatively correlated. It was interesting that different types of saponins showed opposite correlations to the anti-cholesterol activity. More experiments are needed to study their actual effects on anti-cholesterol and the possible underlying mechanisms.

## 4. Conclusion

The research investigated and compared the chemical composition and anti-cholesterol activity of tea flowers from three albino cultivars and one non-albino cultivar. Unlike tea leaves, the content of free amino acids in tea flowers from albino cultivars was not significantly higher than that in tea flowers from the non-albino Jiukeng cultivar and the content of polyphenols was not lower. Among the three albino samples, tea flowers from Yujinxiang had the highest free amino acids, but lowest polyphenols, especially procyanidins. On the contrary, tea flowers from Huangjinya had the highest polyphenols and saponins, but lowest free amino acids. *In vitro* anti-cholesterol activity assays revealed that tea flowers from Yujinxiang exhibited stronger activity in decreasing the micellar cholesterol solubility, while the cholesterol esterase inhibitory activity and bile salt binding activity of the four samples were insignificantly different. Future studies on the investigation of key active anti-cholesterol compounds can be conducted.

## Data availability statement

The data presented in the study are deposited in the MetaboLights repository, accession number MTBLS7012.

## Author contributions

YG, ZH, Y-QX, and J-FY conceived, designed the experiments, and wrote the paper. YG performed the experiments and analyzed the data. All authors contributed to the article and approved the submitted version.

## Funding

This research was supported by the National Natural Science Foundation of China (32202114), China Agriculture Research System of MOF and MARA (CARS-19), and the Innovation Project for the Chinese Academy of Agricultural Sciences.

## Conflict of interest

The authors declare that the research was conducted in the absence of any commercial or financial relationships that could be construed as a potential conflict of interest.

## Publisher’s note

All claims expressed in this article are solely those of the authors and do not necessarily represent those of their affiliated organizations, or those of the publisher, the editors and the reviewers. Any product that may be evaluated in this article, or claim that may be made by its manufacturer, is not guaranteed or endorsed by the publisher.
