# Supplementary material for: Chemical composition and anti-cholesterol activity of tea (Camellia sinensis) flowers from albino cultivars
Source: Front Nutr. 2023 Mar 27;10:1142971. doi: 10.3389/fnut.2023.1142971 (PMC10083420; doi:10.3389/fnut.2023.1142971)
Supplement: Supplementary file 1 [file Data_Sheet_1.docx]

Supplementary Material

Chemical composition and anti-cholesterol activity of tea (*Camellia sinensis*) flowers from albino cultivars

Ying Gao^1^, Zhen Han^2^, Yong-Quan Xu^1*^, Jun-Feng Yin^1*^

*** Correspondence:**

Yong-Quan Xu
yqx33@126.com

Jun-Feng Yin
yinjf@tricaas.com

**Table S1.** Identification of constituents in tea flowers by UPLC-QE-Orbitrap-MS

| RT (min) | Molecular  formula | [M-H]- | | MS/MS Fragments m/z | Tentative identification | Qualitative method |
| --- | --- | --- | --- | --- | --- | --- |
|  |  | Theoretical | Experimental |  |  |  |
| **Organic acids** | |  |  |  |  |  |
| 0.87 | C_7_H_12_O_6_ | 191.055 | 191.0556 | 85.0284 | Quinic acid | Standard |
| 0.96 | C_4_H_6_O_5_ | 133.0131 | 133.0136 | 115.0027; 89.0233; 71.0127 | Malic acid | Standard |
| 1.15 | C_6_H_8_O_7_ | 191.0186 | 191.0189 | 111.0075; 87.0075; 85.0283 | Citric acid | Standard |
| 1.45 | C_4_H_6_O_4_ | 117.0182 | 117.0184 | 99.0079; 73.0285 | Succinic acid | Standard |
| 1.55 | C_7_H_6_O_5_ | 169.0131 | 169.0135 | 125.0234 | Gallic acid | Standard |
| **Procyanidins** | |  |  |  |  |  |
| 4.12 | C_30_H_26_O_12_ | 577.1341 | 577.1361 | 407.0776; 289.0721; 125.0234 | Procyanidin B1 | Standard |
| 5.07 | C_30_H_26_O_12_ | 577.1341 | 577.1359 | 407.0776; 289.0721; 125.0234 | Procyanidin B2 | Standard |
| 5.34 | C_45_H_38_O_18_ | 865.1974 | 865.2004 | 407.0776; 289.0721; 125.0234 | Procyanidin C1 | Standard |
| **Flavonols and flavonol glycosides** | | |  |  |  |  |
| 8.70 | C_15_H_10_O_6_ | 285.0394 | 285.0408 | 151.0027 | Kaempferol | Standard |
| 7.73 | C_15_H_10_O_7_ | 301.0343 | 301.0357 | 178.9980; 151.0028 | Quercetin | Standard |
| 6.18 | C_15_H_10_O_8_ | 317.0292 | 317.0305 | 178.9980; 151.0028; 137.0235 | Myricetin | Standard |
| 5.01 | C_21_H_20_O_12_ | 463.0871 | 463.0887 | 300.0279; 169.0137 | Quercetin-glucoside | Standard |
| 4.76 | C_27_H_30_O_16_ | 609.1450 | 609.1469 | 300.0280 | Quercetin-rutinoside | Standard |
| 4.87 | C_21_H_20_O_12_ | 463.0871 | 463.0886 | 300.0280; 169.0136 | Quercetin-galactoside | Standard |
| 4.27 | C_33_H_40_O_21_ | 771.1978 | 771.2007 | 300.0280 | Quercetin-rutinosyl-galactoside | Tentative |
| 4.47 | C_33_H_40_O_21_ | 771.1978 | 771.2006 | 300.0280 | Quercetin-rutinosyl-glucoside | Tentative |
| 5.44 | C_21_H_20_O_11_ | 447.0921 | 447.0940 | 284.0331 | Kaempferol-galactoside | Standard |
| 5.70 | C_21_H_20_O_11_ | 447.0921 | 447.0940 | 284.0331 | Kaempferol-glucoside | Standard |
| 5.44 | C_27_H_30_O_15_ | 593.1501 | 593.1523 | 284.0331 | Kaempferol-rutinoside | Standard |
| 4.79 | C_33_H_40_O_20_ | 755.2029 | 755.2053 | 593.1521; 284.0331 | Kaempferol-rutinosyl-galactoside | Tentative |
| 5.10 | C_33_H_40_O_20_ | 755.2029 | 755.2054 | 593.1521; 284.0331 | Kaempferol-rutinosyl-glucoside | Tentative |
| 4.00 | C_21_H_20_O_13_ | 479.0820 | 479.0839 | 316.0228 | Myricetin-galactoside | Standard |
| 4.11 | C_21_H_20_O_13_ | 479.0820 | 479.0839 | 316.0228 | Myricetin-glucoside | Standard |
| 4.00 | C_27_H_30_O_17_ | 625.1399 | 625.1421 | 316.0229 | Myricetin-rutinoside | Standard |
| **Saponins** |  |  |  |  |  |  |
| 10.8 | C_59_H_92_O_27_ | 1231.5742 | 1231.5754 | 1099.5334; 1051.5190; 949.4782; 919.4688; 627.3915 | Chakasaponin III | Tentative |
| 11.99 | C_60_H_94_O_26_ | 1229.595 | 1229.5968 | 1083.5435; 1066.5289; 1049.5341; 951.5025; 611.3956 | Floratheasaponin D | Tentative |
| 12.11 | C_59_H_92_O_26_ | 1215.5793 | 1215.5815 | 1083.5439; 1035.5193; 951.5029; 933.4831; 611.3956 | Chakasaponin I | Tentative |
| 12.9 | C_59_H_92_O_26_ | 1215.5793 | 1215.5815 | 1083.5439; 1035.5193; 951.5029; 933.4831; 611.3956 | Floratheasaponin A | Tentative |
| 14.57 | C_62_H_96_O_27_ | 1271.6055 | 1271.6074 | 1110.5637; 1091.5463; 993.5057; 653.4080 | Floratheasaponin H | Tentative |
| 16.19 | C_62_H_96_O_27_ | 1271.6055 | 1271.6075 | 1139.5627; 1091.5439; 1007.5268; 667.4223 | Chakasaponin II | Tentative |
| 16.81 | C_62_H_96_O_27_ | 1271.6055 | 1271.6073 | 1139.5718; 1091.5459; 989.5180; 667.4243 | Floratheasaponin J | Tentative |
| 17.16 | C_63_H_98_O_27_ | 1285.6212 | 1285.6212 | 1123.5702; 1105.5609; 669.4401 | Floratheasaponin E | Tentative |
| 17.24 | C_62_H_96_O_27_ | 1271.6055 | 1271.6074 | 1139.5718; 1091.5458; 1007.5249; 667.4236 | Floratheasaponin B | Tentative |
| 17.33 | C_63_H_100_O_27_ | 1287.6368 | 1287.6370 | 1141.5817; 1107.5771; 1009.5399; 667.4227 | Floratheasaponin F | Tentative |
| 17.38 | C_62_H_98_O_27_ | 1273.6212 | 1273.6227 | 1141.5793; 1093.5596; 1009.5396; 669.4388 | Floratheasaponin C | Tentative |
| **Methylxanthines** | | [M+H]+ | |  |  |  |
| 4.74 | C_8_H_10_N_4_O_2_ | 195.0877 | 195.0877 | 138.0662; 110.0715 | Caffeine | Standard |
| 2.40 | C_7_H_8_N_4_O_2_ | 181.0720 | 181.0721 | 163.0614; 137.0823; 122.0589 | Theobromine | Standard |
| 3.57 | C_7_H_8_N_4_O_2_ | 181.0720 | 181.0721 | 159.9693; 141.9587; 124.0507 | Theophylline | Standard |


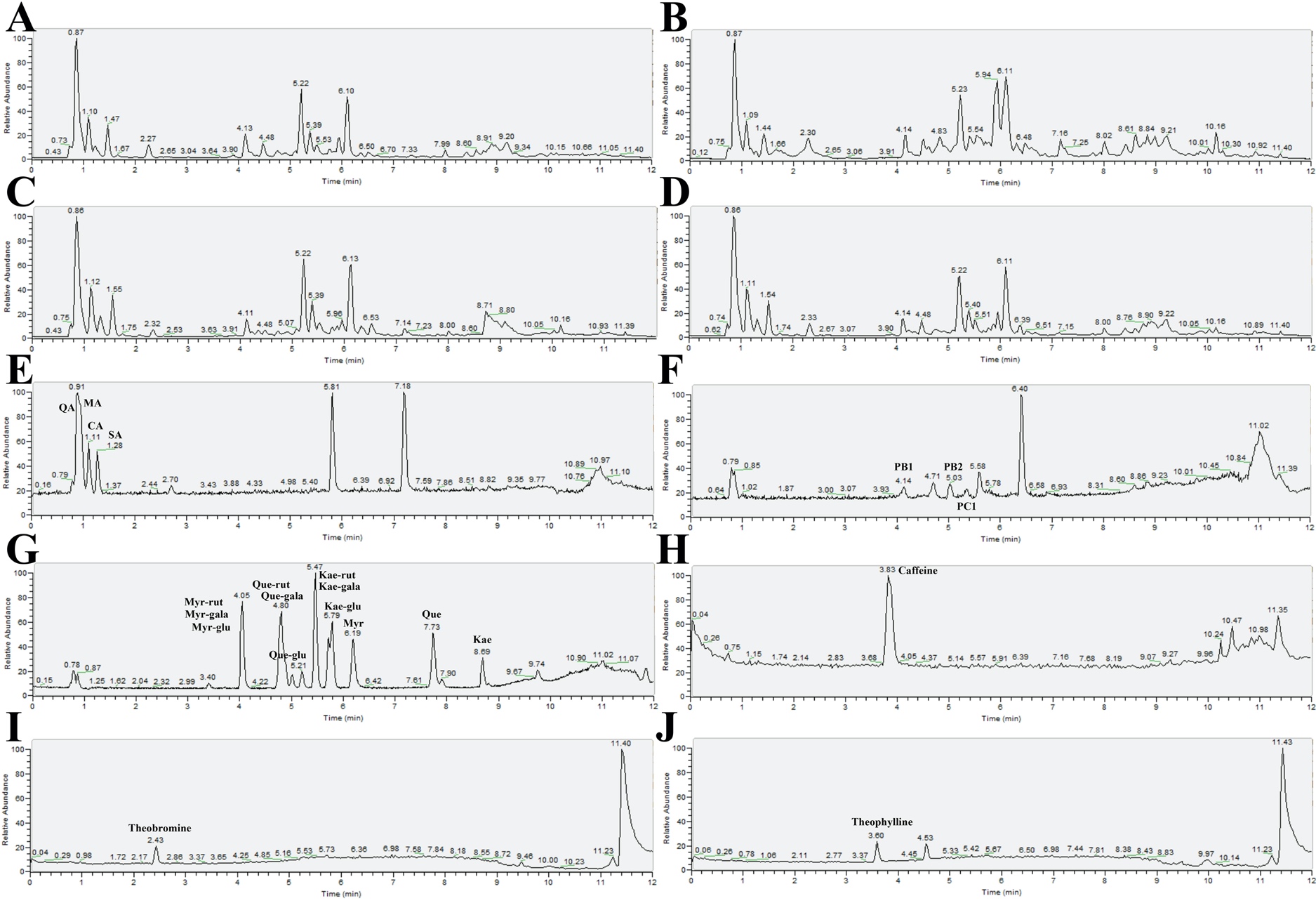


**Figure S1.** Chromatograms of ultrahigh performance liquid chromatography-Q Exactive-Orbitrap-mass spectrometry. (A) Baiye No.1; (B) Huangjinya; (C) Yujinxiang; (D) Jiukeng; (E) standards of organic acids; (F) standards of procyanidins; (G) standards of flavonols and flavonol glycosides; (H) the caffeine standard; (I) the theobromine standard; (J) the theophylline standard.


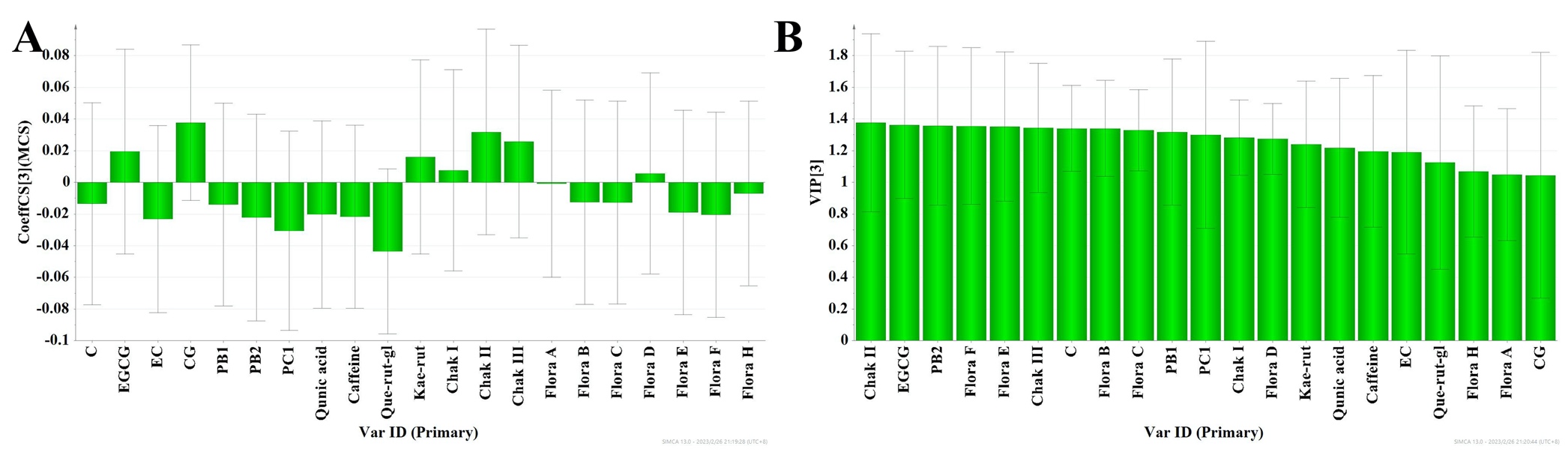


**Figure S2.** Results of partial least squares regression. (A) The coefficient plot; (B) The variable importance in projection plot.

**Supplementary materials & methods**

**1. Determination of total phenolic content**

The total phenolic content was measured according to the national standard GB/T 8313-2008. One milliliter diluted tea flower infusion was mixed with 5 mL 10% Folin & Ciocalteu’s phenol reagent, stayed at room temperature for 6 min, then mixed 4 mL 7.5% Na_2_CO_3_ aqueous solution, stayed at room temperature for 1 h, and read the absorbance at 765 nm in a 1 cm path length cuvette using a UV-VIS-NIR spectrophotometer (UV-3600, Shimadzu Co., Ltd., Kyoto, Japan).

**2. Determination of eight monomeric catechins**

The contents of eight monomeric catechins were measured using a HPLC method. The infusion was filtered through a Millipore filter (0.45 μm) before injection. The injection volume was 10 μL. The separation was performed on a Diamonsil^TM^ C18 column (5 μm, 4.6 mm × 250 mm, Dikma Technologies Inc., Lake Forest, CA, United States) using a Shimadzu Prominence LC-20A system (Shimadzu (Suzhou) Corporation, Suzhou, China). A 2% acetic acid in water and acetonitrile was used as mobile phases A and B. The gradient changes of mobile phases were 0–16 min, 6.5–15% B; 16–25 min, 15–25% B; 25–25.5 min, 25%; 25.5–30 min, 25–6.5% B; 30–35 min, 6.5% B. The total flow rate was 1 mL/min. The column temperature was 35℃. The absorbance at 280 nm was monitored using a Shimadzu SPD-10A ultraviolet detector (Shimadzu (Suzhou) Corporation).

**3. Determination of free amino acids**

The content of free amino acids was measured according to the national standard GB/T 8314-2013. One milliliter tea flower infusion was mixed with 0.5 mL 2% ninhydrin and 0.5 mL 1/15 mol/L phosphate buffered saline (pH 8.0), incubated at 100℃ for 15 min, cooled to room temperature, added water to reach a total volume of 25 mL, stayed for 10 min, and read the absorbance at 570 nm in a 0.5 cm path length cuvette using a UV-VIS-NIR spectrophotometer (UV-3600, Shimadzu Co., Ltd., Kyoto, Japan).

**4. Determination of the composition of free amino acids**

The composition of free amino acids was measured using an amino acid analyzer. Five milliliters of infusion were evaporated and the residue was redissolved in 0.02N HCl solution. The infusion was filtered through a Millipore filter (0.22 μm) before injection. The injection volume was 50 μL. The separation was performed on a LCA K07/Li cation separation column (7 μm, 4.6 mm × 150 mm) using a Sykam amino acid analyzer (Sykam S433-D, Eresing, Germany). Li-citrate/borate buffer A (0.12 N, pH 2.95), Li-citrate/borate buffer B (0.3 N, pH 4.20), Li-citrate/borate buffer C (0.3 N, pH 8.00), and regeneration solution (0.45 N) were used as mobile phases A, B, C, and D. The gradient changes of mobile phases were 0–10 min, 100% A; 10–11 min, 100–79% A, 0–21% B; 11–30 min, 79% A, 21% B; 30–41 min, 79–62% A, 21–38% B; 41–63 min, 62–0% A, 38–100% B; 63–68 min, 100–0% B, 0–100% C; 68–78 min, 100% C; 78–81 min, 100–86% C, 0–14% D; 81–83 min, 86% C, 14% D; 83–95 min, 78–76% C, 22–24% D; 95–101 min, 76% C, 24% D; 101–106 min, 100% D; 106–130 min, 100% A. The total flow rate of mobiles phases was 0.45 mL/min. The flow rate of ninhydrin solution was 0.25 mL/min. The reaction temperature was 130℃. The absorbance at 570 nm and 440 nm were monitored.
